# Acute respiratory infections in an adult refugee population: an observational study

**DOI:** 10.1038/s41533-021-00261-9

**Published:** 2021-12-21

**Authors:** Alexandra Jablonka, Christian Dopfer, Christine Happle, Andree Shalabi, Martin Wetzke, Eva Hummers, Tim Friede, Stephanie Heinemann, Nele Hillermann, Anne Simmenroth, Frank Müller

**Affiliations:** 1 German Center for Infection Research (DZIF), Partner site Hannover-Braunschweig, Hannover, Germany; 2 Department of Rheumatology and Immunology, Hannover Medical School, Hannover, Germany; 3 Department of Pediatrics, Neonatology and Allergology, Hannover Medical School, Hannover, Germany; 4 Department of General Practice, University Medical Center Goettingen, Goettingen, Germany; 5 Department of Medical Statistics, University Medical Center Goettingen, Goettingen, Germany; 6 Department of General Practice, University Hospital Wuerzburg, Wuerzburg, Germany

**Keywords:** Health occupations, Respiratory signs and symptoms, Epidemiology

## Abstract

The presence of acute infectious respiratory diseases (ARD) is one of the main reasons why recently arrived refugees seek medical help. This paper investigates the incidence rates of acute respiratory diseases in an adult refugee population as well as associated sociodemographic factors and drug treatments. We conducted a retrospective observational study of deidentified medical records. The data were collected between 2015 and 2019 in the health care centers of two large German initial reception centers for refugees. Multivariable analyses controlling for sociodemographics were carried out using generalized estimating equations. Out of 10,431 eligible residents, 6965 medical encounters of 2840 adult patients were recorded over 30 months. Of all the adult patients, 34.4% sought medical help for a respiratory symptom or diagnosis at least once. Older patients and patients from Sub-Saharan Africa sought help less often. The occurrence of ARD showed a typical distribution over the course of the year. Facility occupancy was not associated with ARD occurrence. Acute respiratory symptoms are a leading cause for adult refugee patients to seek medical care. The doctor contact rates due to ARD were consistently two to three times higher among refugees than among German residents.

## Introduction

Refugees are at particular risk for transmittable diseases for a variety of reasons^1^. The circumstances of their journey often make it impossible to take the simplest measures for hygiene, such as regular hand washing. Refugees regularly flee in smaller or larger groups, many live in extremely confined conditions or in refugee camps with many other people sharing a small space. In the countries of arrival, they are often housed in collective accommodations that seem to facilitate the spread of infectious and parasitic diseases^2^. High prevalence rates of scabies infestation^3^, reports of outbreaks of measles^4,5^ and varicella^6–8^ and protozoa infections^9^ have been described in this population. Acute respiratory infections are among the most common reasons for seeking primary medical care among refugees, migrants, and nonmigrants in Germany^10^.

Currently, the global severe acute respiratory syndrome coronavirus 2 (COVID-19) pandemic poses a particular risk to marginalized populations^11^. Initial findings suggest that these populations are overrepresented in terms of prevalence and mortality^12^. This is particularly true for refugees and migrants^13^. Germany has taken in over 2.3 million people in the last decade, the majority of whom were seeking protection from civil wars in the Middle East^14,15^. It has been widely acknowledged that health care systems need to include and address these groups^5–7^, and this is especially important with regard to the successful mitigation of the COVID-19 pandemic.

In addition, various studies have shown that respiratory diseases are not only very common among recently arrived refugees and migrants^16^ but are the most common reason why newly arrived refugees and migrants seek medical assistance^17,18^.

This study aims to provide insights into the incidence of respiratory diseases in adult refugees, their distribution over time, factors influencing their development, and the treatment regimens chosen. Subsequently, we would like to discuss, how the results of this study can inform policies regarding the COVID-19 screening of newly arrived refugees and migrants.

## Methods

### Settings

We collected data from two refugee cohorts in two different locations in Northern Germany. One location was the medical ward of the reception facility for newly arriving refugees in Celle. Data were collected between September 2015 and June 2016. This facility was only temporarily in use to deal with peak numbers of refugees in 2015. It was closed in June 2016, when the number of refugees declined. Some aspects of this cohort have been described in previous studies^19^.

The second cohort was located in Friedland. Data were collected in a similar medical ward of a reception facility for newly arrived refugees between August 2017 and January 2019. A subsample of the Friedland cohort has been previously described^20,21^.

Asylum seekers were accommodated in one of the two locations as soon as they entered Germany and claimed asylum. They were accommodated only for a transitional period of weeks up to months. By applying a national distribution plan called “Königssteiner Schlüssel,” asylum seekers were then distributed to other municipalities all over Germany.

The services of these facilities (run by the regional government of Lower Saxony in Northern Germany) included accommodation, meals, social services, and medical services. Primary medical care was provided on site according to the German “asylum seeker benefits law” (Asylbewerberleistungsgesetz). This includes only basic health care, but patients can also be referred to specialist treatments or to hospitals if needed. Emergency medical personnel are available 24 h each day of the week, and consultation hours in the medical ward are provided by a number of medical doctors during the day, but not on weekends. Refugees and migrants can receive medical assistance exclusively through onsite medical wards.

### Data collection and management

This observational study uses depersonalized data obtained by a retrospective chart review of diagnoses (coded as International Statistical Classification of Diseases and Related Health Problems [ICD-10]) and prescribed medications (coded as ATC, Anatomical Therapeutic Chemical Classification System) of each consultation in the aforementioned medical wards. The study adheres to the STROBE statement. We extracted basic demographic information from the chart of each patient, including age, sex, refugee status, nationality, and whether patients received treatment from a general practitioner or a specialist. The review was performed by one medical student (N.H.) under the supervision of experienced medical doctors. N.H. was trained prior to coding, and diagnoses were regularly reviewed. Diagnoses were classified post hoc. The coder was instructed to also code individual symptoms, such as fever (e.g., as ICD-10 code R50), if this was noted in the patient records. Minors aged 0–18 years were excluded from the analysis.

### Definitions

For the analyses, we differentiated between four different groups of respiratory ailments that were identified by analyzing the diagnostic ICD-10 codes issued by the onsite physicians. These were (a) acute respiratory diseases (ARD), which include common colds, bronchitis, and typical symptoms thereof, and (b) influenza-like illness (ILI), which was coded when the fever was diagnosed in addition to an ARD diagnosis. The diagnosis of (c) pneumonia was separately issued, as this condition can be seen as a complication of ARD/ILI and some chronic respiratory diseases (CRD), such as chronic obstructive pulmonary disease. (d) CRD were used to cover diseases with symptoms lasting >6 weeks or with a chronic recurrent course. Thus, a patient with acute bronchitis would be categorized as having ARD; if fever would accompany it, it would be an ILI. A detailed list of ICD-10 codes that qualify for the categories used here is provided in Supplementary Table 1. The diagnoses were issued upon the judgment of the treating physician, who were primarily experienced general practitioners. Frequently, patients with chronic conditions have previous reports with them, which often support the clinical picture. In addition, mandatory chest X-rays are taken as part of the initial admission. In the case of abnormalities in these X-rays, patients are referred to the onsite physician. In the case of serious illnesses (e.g., episode of an asthma bronchiale), patients are referred to hospital or specialist practices. The resulting diagnoses were then recorded during the chart review and therefore included in the study. Spirometry was only available upon referral, therefore, most diagnoses were based upon clinical judgment or patients’ medical history.

The definitions of ARD and ILI are based upon the classification of the Robert Koch Institute, the German National Institute of Public Health^22^. By using this classification, observational data from domestic cohorts can be used for comparison.

The “country of origin” was coded according to official documents issued to refugees and asylum seekers from the local government as part of the legal asylum claims process. We used the World Bank Regional Groupings according to the Atlas of Sustainable Development Goals 2018 to describe a patient’s region of origin^23^. A chronic condition was assessed by using the “Chronic Condition Indicator for ICD-10-CM of the fiscal year 2020” by the Agency for Healthcare Research and Quality. Any ICD-10 diagnoses given to patients were checked if they matched the criteria of a chronic condition^24^.

The use of the term “refugees” has been recommended by the United Nations High Commissioner for Refugees (UNHCR) to differentiate officially recognized refugees from migrants without this status^3,4^. Asylum seekers, who are not yet officially recognized as refugees, are subsumed as “refugees” in this study. In addition to asylum seekers, our study includes a second category of refugees called “resettlement refugees.” Resettlement refugees have been recognized by the UNHCR as particularly vulnerable since they neither return to their home country nor stay in the country to which they have fled. As part of multilateral agreements, resettlement refugees are permanently resettled in a third country. Typically, resettled refugees are selected by UNHCR staff and safely transferred to their country of destination in larger groups. Data concerning the healthcare needs of resettled refugees from parts of this cohort have already been published^16^.

### Statistical analyses

We used descriptive statistics including absolute and relative frequencies, medians, and interquartile ranges (IQRs) to describe the distribution of respiratory diseases in relation to sociodemographic factors. We also characterized the population by summarizing data at the individual patient level. Patients with and without respiratory diseases were compared according to their age and the frequency of consultations. Since both of these dependent variables were non-normally distributed, Mann–Whitney *U*-test was used to test for significant differences.

We conducted a multivariable analysis using logistic regression models to determine factors that might impact the likelihood of a diagnosis of respiratory disease in this setting. The literature shows that shelter occupancy plays a role in the development of respiratory infections in refugee children^20^. Furthermore, refugee status (refugee or resettlement refugee) has a relevant impact on health care utilization^16^. Many acute respiratory infections have a seasonal peak in fall^25^, and children are more often affected than adults^26^. These variables, collected as part of the study, were therefore included in the regression model. Additionally, patients’ age group, sex, and region of origin were considered relevant sociodemographic confounders and were therefore included in the regression model.

We corrected the standard errors of the regression coefficients by using generalized estimating equations (GEEs). This approach also considers that patients can have several consultations. Differences in drug prescriptions were tested using Pearson’s chi-square test for categorical variables, e.g., with patients’ sex, or Mann–Whitney *U* for metric variables, e.g., patients’ age. For all calculations, *p* values (*p*) ≤0.05 were considered statistically significant. Statistical analyses were performed using SPSS (Version 25, IBM, Armonk, NY, USA), and figures were plotted using a Google Spreadsheet (Alphabet Inc., Mountain View, CA, USA).

### Power and sample size

An estimated sample size of *n* = 2300 patients was determined to be sufficiently large to detect relevant effect sizes in this population. For instance, the total sample size split in a 5:1 ratio (i.e., *n* = 1917 vs. *n* = 383 patients) provides a power of 95% at a two-sided significance level of 5% given a group difference in airway infections of 50 vs. 60%. Power and sample size calculations were carried out with ClinCalc^27^.

### Ethics compliance

This study was approved by local authorities (Institutional Review Board of Hannover Medical School approval No. 3217–2016, Research Ethics Board of the University Medical Center Goettingen approval No. 16/3/17 as part of the DICTUM Friedland study^28^). Obtaining patients’ written informed consent was not necessary for this study. The data were collected by performing a retrospective chart review and processed in a pseudonymized manner. The decision to waive obtaining informed consent was approved by both responsible ethics committees.

## Results

**Fig. 1:**
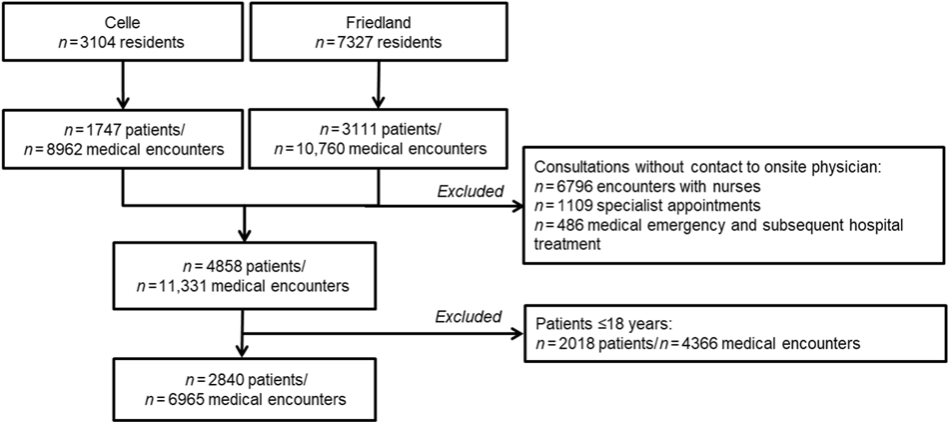
Flow chart of patient inclusion.

Altogether, 10,431 refugees, including asylum seekers and resettled refugees (7327 in Friedland, 3104 in Celle), were accommodated during the observational period (Fig. 1). The study included 2840 adult refugee patients. Overall, we report data spanning an observational period of 2.5 years including the fall/winter season 2015/2016 (Celle), 2017/2018 (Friedland), and 2018/2019 (Friedland). The occupancy rates in both camps ranged from 1 to 99% during all study dates, with a median occupancy of 39.7% (30%).

### Demographics of adult patients

This analysis included adult patients only (*n* = 2840). The median age was 32 years old (IQR 15), and 53.7% (*n* = 1526) were male (Supplementary Fig. 1). Patients mainly came from Syria (*n* = 981, 34.5%), Iraq (*n* = 422 14.9%), and Afghanistan (*n* = 292, 10.3%). A total of 76.9% (*n* = 2184) of patients were refugees, including asylum seekers, while 656 were resettled refugees. We counted a total of 6965 consultations, where 9533 diagnoses were given. The median number of consultations with doctors was 2 (min: 1, max: 44, IQR 2). Of all 2,840 adult patients, 34.4% (*n* = 969) had at least one respiratory diagnosis (ARD or signs of it, ILI, pneumonia, or CRD) (Table 1). In 19.7% (*n* = 1209) of 6965 recorded consultations, respiratory symptoms were diagnosed (Table 2).

**Table 1:** Characteristics of patients with and without a respiratory diagnosis (ARD, ILI, pneumonia, CRD).

Characteristic		Patients without any respiratory diagnosis (*n* = 1871)	Patients with respiratory diagnosis (*n* = 969)	*p*^a^
		*n* (%)	*n* (%)	
Sex^b^	Female	868 (67.3)	442 (33.7)	0.657
	Male	999 (65.5)	527 (34.5)	
Age (years)	19–29	729 (65.6)	382 (34.4)	0.117
	30–39	568 (64.1)	318 (35.9)	
	40–49	306 (66.5)	154 (33.5)	
	50–59	150 (68.2)	70 (31.8)	
	60–69	87 (68.5)	40 (31.5)	
	70+	31 (86.1)	5 (13.9)	
Region of origin^c^	Europe & Central Asia	247 (74.0)	87 (26.0)	<0.001
	Latin America & Caribbean	17 (85.0)	3 (15.0)	
	Middle East & North Africa	1072 (62.7)	638 (37.3)	
	South & East Asia	218 (59.2)	157 (40.8)	
	Sub-Saharan Africa	222 (79.6)	57 (20.4)	
	Unknown/stateless	57 (75.0)	19 (25.0)	
Reception facility	Celle	477 (49.5)	486 (50.5)	<0.001
	Friedland	1394 (74.3)	483 (25.7)	
Refugee category	Asylum seeker	1443 (66.1)	741 (33.9)	0.695
	Resettlement	428 (65.2)	228 (34.8)	
Chronic condition present	None	1430 (64.0)	806 (36.0)	<0.001
	Any	441 (73.0)	163 (27.0)	
	Hypertension	122 (73.5)	44 (26.5)	0.002
	Diabetes	47 (75.8)	15 (24.2)	0.026
	Coronary heart disease	23 (65.7)	12 (34.3)	0.811

^a^Pearson’s chi-square test.

^b^Missing *n* = 4.

^c^Missing *n* = 46.

**Table 2:** Characteristics of consultations.

Characteristic		All onsite consultations (*n* = 6965)	Consultations with patients with acute respiratory diseases or signs thereof (ARDs) (*n* = 1209)			
		*n* (%)	*n* (%)	% proportion	Multivariable OR^a^	*p*
Sex^b^	Female	3417 (49.1)	556 (16.3)	46.0	Ref.	0.687
	Male	3540 (50.9)	653 (18.4)	54.0	1.03 (0.88–1.21)	
Age (years)	19–29	2684 (38.5)	516 (19.2)	42.7	Ref.	0.003
	30–39	2068 (29.7)	385 (18.6)	31.8	1.08 (0.90–1.30)	
	40–49	1160 (16.7)	193 (16.6)	16.0	0.92 (0.72–1.17)	
	50–59	633 (9.1)	77 (12.2)	6.4	0.75 (0.55–1.03)	
	60–69	324 (4.7)	34 (10.5)	2.8	0.63 (0.42–0.95)	
	70+	96 (1.4)	4 (4.2)	0.3	0.18 (0.05–0.60)	
Region of origin^c^	Europe & Central Asia	921 (13.3)	87 (9.4)	7.2	Ref.	0.018
	Latin America & Caribbean	28 (0.4)	3 (10.7)	0.2	1.75 (0.46–6.64)	
	Middle East & North Africa	3785 (54.7)	759 (20.1)	63.2	1.03 (0.76–1.40)	
	South & East Asia	1371 (19.8)	280 (20.4)	23.3	1.09 (0.78–1.54)	
	Sub-Saharan Africa	671 (9.7)	56 (8.3)	4.7	0.58 (0.38–0.87)	
	Unknown/stateless	144 (2.1)	16 (11.1)	1.3	0.86 (0.46–1.60)	
Reception facility	Celle	3203 (46.0)	759 (23.7)	62.8	Ref.	<0.001
	Friedland	3762 (54.0)	450 (12.0)	37.2	0.44 (0.34–0.55)	
Occupancy of facility	0–20%	332 (4.9)	45 (13.6)	3.8	1.57 (1.03–2.40)	0.110
	20–40%	1892 (27.9)	232 (12.3)	19.4	Ref.	
	40–60%	2360 (34.8)	379 (16.1)	31.8	1.09 (0.89–1.34)	
	60–80%	1693 (24.9)	404 (23.9)	33.9	1.30 (1.00–1.69)	
	80–100%	514 (7.6)	133 (25.9)	11.1	1.15 (0.83–1.59)	
Month of treatment	January	1009 (14.5)	180 (17.8)	14.9	Ref.	<0.001
	February	715 (10.3)	174 (24.3)	14.4	1.47 (1.11–1.95)	
	March	568 (8.2)	106 (18.7)	8.8	1.21 (0.83–1.76)	
	April	457 (6.6)	45 (9.8)	3.7	0.59 (0.37–0.95)	
	May	296 (4.2)	28 (9.5)	2.3	0.47 (0.24–0.89)	
	June	182 (2.6)	18 (9.9)	1.5	0.66 (0.35–1.25)	
	July	194 (2.8)	21 (10.8)	1.7	0.94 (0.49–1.78)	
	August	402 (5.8)	33 (8.2)	2.7	0.86 (0.53–1.39)	
	September	875 (12.6)	160 (18.3)	13.2	1.28 (0.95–1.72)	
	October	848 (12.2)	178 (21)	14.7	1.35 (0.99–1.85)	
	November	693 (9.9)	87 (12.6)	7.2	0.90 (0.62–1.29)	
	December	726 (10.4)	179 (24.7)	14.8	1.70 (1.25–2.31)	
Refugee category	Asylum seeker	6133 (88.1)	1006 (16.4)	83.2	Ref.	<0.001
	Resettlement	832 (11.9)	203 (24.4)	16.8	3.03 (2.33–3.95)	
Chronic condition present	No	4599 (66)	994 (21.6)	82.2	Ref.	<0.001
	Yes	2366 (34)	215 (9.1)	17.8	0.45 (0.37–0.54)	

^a^Multivariable regression analyses: confidence intervals and test for significance were adjusted by robust estimates.

^2^Missing *n* = 8.

^3^Missing *n* = 45.

### Acute respiratory diseases

The most common ARD diagnoses were sore throat (R07.0, in *n* = 476, 5.0% of all diagnoses), cough (R05, *n* = 377, 4.0% of all diagnoses), cold (J00, *n* = 281, 2.9% of all diagnoses), and unspecified acute upper respiratory infection (J06.9, *n* = 227, 2.4% of all diagnoses).

There were no significant differences for adult patients with an ARD diagnosis in comparison to patients without such a diagnosis in terms of sex (male sex with ARD 54.5% vs. without ARD 53.5%, *p* = 0.643) and refugee status (refugee with ARD 77.8% vs. without ARD 76.5%, *p* = 0.476). However, patients with an ARD diagnosis received significantly more treatments (median 2 [IQR 4, mean 3.7] vs. 2 [IQR 2, mean 2.5], *p* < 0.001) and were slightly younger (median 32 [IQR 14] years vs. 33 [IQR 16] years, *p* = 0.020). In 16% of cases, patients with an ARD diagnosis also had a chronic condition.

### ILI and pneumonia

ILI was diagnosed in 2.6% (*n* = 74) of all adult patients. Patients with ILI did not significantly differ from other patients concerning age (median age of ILI patients 31 years [IQR 12] vs. 32 years [IQR 16], *p* = 0.068) and refugee status (83.8% asylum seeker in ILI group vs. 76.7% asylum seeker in the non-ILI group, *p* = 0.155). However, patients with ILI were significantly more often male (66.2% male with ILI vs. 53.5% male without ILI, *p* = 0.030) and had more consultations (median 3 [IQR 4] in ILI vs. 2 [IQR 2] in non-ILI, *p* = 0.001).

Pneumonia patients (*n* = 23; 0.9%) had a median age of 36 years (IQR 20) compared to slightly younger nonpneumonia patients (33 years [IQR 16], *p* = 0.531).

### Chronic respiratory disease

In 55 adult patients (1.9%), chronic respiratory ailment (CRD) was diagnosed. The most common diagnosis was bronchial asthma (J45, *n* = 37 diagnoses), followed by chronic obstructive pulmonary diseases (J44, *n* = 20 diagnoses) and allergic rhinitis due to pollen (J30, *n* = 8). Patients with a CRD did not significantly differ from those without such a diagnosis concerning sex (63.6% male sex in CRD vs. 53.2% male sex in non-CRD, *p* = 0.125) and refugee status (81.8% refugee in CRD vs. 77.0% refugee in non-CRD, *p* = 0.398). However, patients with a CRD were older (median age 38 years [IQR 21] in CRD patients vs. 32 years [IQR 15] in non-CRD patients, *p* = 0.010) and had more consultations with doctors (median 3 [IQR 4] consultations in CRD patients vs. median 2 [IQR 3] in non-CRD patients, *p* < 0.001).

### Distribution of respiratory diseases over time

**Fig. 2:**
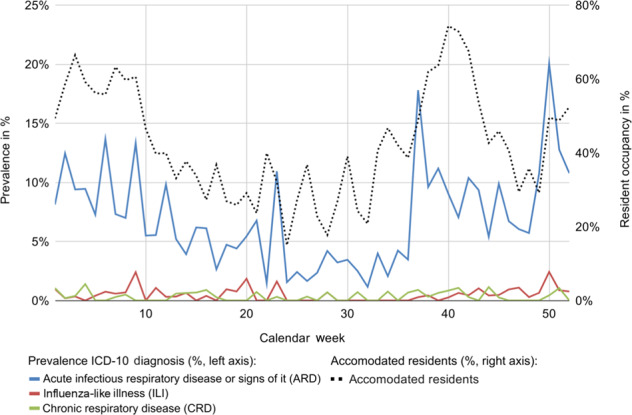
Prevalences of respiratory diseases over the calendar weeks of a year.

In absolute numbers, ARD occurred mostly during fall and winter between September and February, with most cases in January (*n* = 180) and December (*n* = 179). The proportion of ARD cases in all treatments ranged between 24.7% in December and 8.2% in August. In weeks 37 and 50, the highest prevalence rates for ARD were 17.7 and 20.0%, respectively. ILI and CRD were almost equally distributed over the course of a year, with prevalence rates under 2.5% (Fig. 2).

### Regression analyses

We performed logistic regression analyses using GEEs with the presence of ARD as a reason for consultation as a dichotomous dependent variable (Table 2). Independent variables were chosen on a theoretical framework.

The main effects for each independent variable were calculated. All variables were significant in the overall model, except “patients’ sex” and “occupancy of facility.” A similar model with patient age and occupancy of the facility as continuous variables is shown in Supplementary Table 2.

The results revealed that patient age played a considerable role in terms of which patients presented themselves with ARD. Compared to 19–29-year-old patients, patients in their 60s and 70s had significantly fewer of these diagnoses (odds ratio (OR) 0.63 [confidence interval CI 0.42–0.95] and 0.18 [CI 0.05–0.60], respectively). Patients from Sub-Saharan Africa were less likely to seek medical help (OR 0.58 [CI 0.38–0.87]) due to ARD than patients from Europe & Central Asia. As expected, there was a reduction in respiratory diseases in the summer months: contracting such a disease in May was half as likely as in January (OR 0.47 [CI 0.24–0.89]). Patients with chronic conditions were less likely to seek medical help with ARD complaints (OR 0.45 [CI 0.37–0.54]), but resettlement refugees were three times more often affected by ARD than refugees (OR 3.03 [CI 2.33–3.95]). Additionally, the Friedland facility had lower frequencies of patients with ARD (OR 0.44 [CI 0.34–0.55]) than the Celle facility. The OR of ARD was higher in low camp occupancy (0–20%) compared to somewhat higher occupancy (20–40%); however, the overall factor “occupancy of facility” was not tested significantly in our model (*p* = 0.110).

### Drug treatment of ARD

**Fig. 3:**
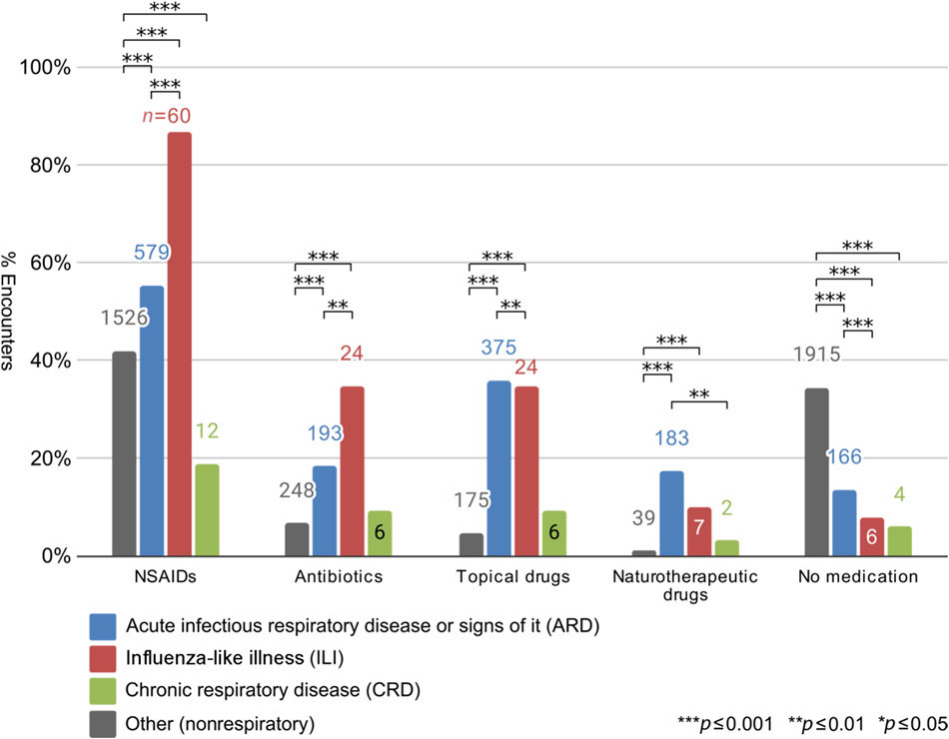
Drug prescriptions in encounters in patients with respiratory and nonrespiratory diagnoses.

Doctors prescribed patients with respiratory ailments mainly nonsteroidal anti-inflammatory drugs (*n* = 579, 55.5%; Table 3). In 18.5% of the ARD cases, antibiotics were prescribed. Patients with ILI received nonsteroidal anti-inflammatory drugs (NSAIDs) and antibiotics significantly more often than patients with ARD. In contrast, patients with ARD received significantly more topical drugs or no prescription at all. More details are shown in Fig. 3.

**Table 3:** Prescriptions in ARD encounters.

	Prescription in ARD encounter		ARD patients’ median age (IQR)		
	*n*	Percent (%)	Received prescription	Other	*p*^a^
No prescription	166	13.7	31.5 (14)	31 (15)	0.949
NSAID	579	55.5	32 (14)	30 (14)	0.012
Ibuprofen	396	32.8			
Paracetamol	133	11.0			
Metamizole	50	4.1			
Aspirin	8	0.7			
Antibiotics	193	18.5	35 (17)	31 (14)	0.001
Amoxicillin	77	6.4			
Doxycycline	32	2.6			
Cefuroxime	16	1.3			
Azithromycin	14	1.2			
Topical drugs	375	36.0	28 (12)	33 (16.5)	<0.001
Benzocaine	207	17.1			
Xylometazoline	174	14.4			
Chlorhexidine	20	1.7			
Ambroxol	12	1.0			
Naturotherapeutic drugs^b^	183	17.5	29 (15)	32 (15)	0.015

^a^*p* values were calculated using Mann–Whitney *U*-test.

^b^All naturotherapeutic substances consisted of Hederae helicis folium [ivy leaf extract].

## Discussion

The objective of the present study was to assess the incidence of different respiratory diseases, such as ARD, ILI, and CRD, in a large cohort of >10,000 refugees and resettlement refugees collected over several years in Northern Germany. Respiratory symptoms were very common: 34.4% of all refugee patients had a respiratory diagnosis during their stay, but only 2% of patients were diagnosed with a CRD. The frequency of ARD diagnoses was higher in winter and in younger patients.

Comparing the rate of consultation due to ARD to findings of the German Federal Epidemiology Reports on influenza and airway infections, in some calendar weeks, our cohort showed two to three times higher rates than the German population. In the German population, the highest frequency of consultations due to ARD in the influenza season between 2001 and 2018 was 2800 visits per 100,000 residents (calendar week 5, 2015)^29^. However, in our adult refugee cohort, we found considerably more contacts per 100,000 residents, e.g., >10,000 ARD consultations in the last three weeks of December with a high of 15,026 contacts per 100,000 residents in calendar week 50. Since we used the same criteria for ARD, a bias seems unlikely here. These findings demonstrate that newly migrated refugees and asylum seekers seek medical help for ARD considerably more often than the nonrefugee German population. This does not necessarily reflect the burden of disease, as care-seeking behavior can be influenced not only by medical factors but also by cultural or social factors^30,31^. Care-seeking behavior in refugees is also influenced by the length of stay and whether a refugee is new to a facility—consultation rates were highest in the first week of stay at the refugee camp^19^. This could indicate that many refugees already came to the facility with an ARD.

Reasons for these findings may lie in the young age of our cohort: The mean age of adults seeking medical care was 35 years, which is widely in range with the overall distribution of adult refugees and migrants, with mainly young males migrating. Males were not more susceptible to ARD but experienced ILIs more frequently than women. This is in line with previous findings that women might be less susceptible to severe respiratory infections, such as influenza and COVID-19^32,33^.

Refugees and migrants often experience an arduous flight and precarious living and housing circumstances, which might affect their nutrition status and immune system. These factors may make refugees more susceptible to infections in general. Precarious living circumstances may also enhance the spread of viral airway infections. However, unlike what we have previously reported for children^20^, no significant association was found between facility occupancy and ARD in adults.

Friedland had lower frequencies of patients reporting ARD symptoms than Celle. This might be due to housing conditions. In Celle, refugees were mainly housed in multiperson tents. In contrast, Friedland provides housing in regular buildings as well as an increased number of sanitary facilities per resident. These environmental factors might have affected the transmission of airborne infections.

Additionally, the Celle facility was operated during a time span, when mainly families with children were seeking shelter^34^. In contrast, the Friedland facility also accommodated single-person refugees. As the prevalence of ARD is much higher among children, it can be assumed that parents and families are more likely to become coinfected^26^. The same can be considered for resettlement refugees, who were typically resettled with greater family networks^35^. Resettlement refugees were three times more likely to seek medical advice for ARD.

Sub-Saharan African refugees were less likely to seek medical help due to ARD. This might be because smoking is generally less common in this region^36,37^, however, smoking status was not assessed in this study.

When analyzing the distribution of respiratory diseases over time, a peak distribution of ARD between September and February was observed. This coincides with the colder seasons in Germany and is known to be associated with increased ARD and ILI rates^25^.

As expected, patients with ILI received NSAIDs and antibiotics significantly more often than patients with ARD and no fever. Patients with ARD received significantly more topical drugs (or no prescription at all) than patients with ILI. Nevertheless, the number of antibiotics prescribed for the treatment of ARD was high, considering that in such cases, the prescription of antibiotics in ARD is not required but can pose an unnecessary risk of adverse effects and harm to patients^38^. In addition, the prescription of antibiotics for ARD can promote microbial resistance.

Microbial resistance is higher in refugees and migrants than in northern European populations^39^. High prescription rates might worsen this problem^40^. In addition, antibiotics may cease to work as expected due to resistance. Nevertheless, antibiotic prescription rates are high not only in refugees but also in the general population. High antibiotic prescription rates can be targeted by patient education^41^. Even so, the implementation of patient education interventions in refugee populations is significantly hindered by language and cultural barriers. This finding could be a starting point for further investigations of antibiotic prescribing in the cohort of refugees.

Only a small number of adult patients presented with a CRD, such as bronchial asthma, chronic obstructive pulmonary diseases, or allergic rhinitis due to pollen. The low prevalence of CRD could possibly be explained by the combination of the young age of the refugees and reduced awareness of CRD, particularly in lower-resource settings^42,43^. This finding is also in line with anecdotal findings from Celle, where refugees were asked about allergies at the camp entrance and only very few refugees reported an allergy. Additionally, a considerable number of individuals were unfamiliar with the disease concept of allergies.

Taking into account that 30% of our refugee population sought out medical care due to respiratory symptoms, the COVID-19 pandemic poses a major threat to refugee populations. Overcrowding, low hygienic standards, and dire living conditions make refugees vulnerable to respiratory diseases, including COVID-19. Equitable access to vaccines should be the goal^44,45^. When refugee camps are experiencing outbreaks, the spread can be very rapid^46^. Screening of newly arrived refugees for COVID-19 may take the peak distribution of ARD into account, as numbers of COVID-19-positive tested patients are still on the rise at the time of writing. High prevalence rates for ARD found with increased numbers of residents strongly suggest the need to strictly implement measurements such as hand hygiene and distance rules to avoid infections, especially with COVID-19^47^. As such, refugee camps should not be overcrowded. Our data show that health care utilization is high when services are offered. These encounters should be used to ensure high vaccine uptake to protect this vulnerable population^45,48^.

One limitation of this study is the quality of information regarding the demographics and age of the refugees. These were obtained by documents presented by refugees and may be inaccurate. For example, there may be certain incentives to get registered as an unaccompanied minor, as this category of a refugee is typically recognized as requiring special protection. Additionally, refugees sometimes do not know their exact date of birth due to missing documents. Furthermore, in some regions of the world, the concept of age in counted years from birth is not widespread and public birth registers are lacking, so approximate ages are stated upon immigration.

Diagnoses and prescribed medication were classified post hoc, possibly introducing a categorization bias. To limit systematic coding errors, the coder was carefully selected, trained, and supervised, and the same person coded the entire dataset.

We excluded visits to nurses in our study because, unlike in other countries, nurses in Germany do not carry out treatments themselves but are involved in handing out prescribed medications, changing bandages, etc. If nurses are confronted with acute treatment requests, patients are typically referred to the physicians on duty.

Refugees with respiratory symptoms may have refused treatment, albeit to a small extent, which may have led to an underestimation of the incidence of respiratory disease. Hospital treatments were also excluded, but these were very rare, and their diagnoses could be considered in follow-up visits to physicians.

The rate of ARDs could also be influenced by the length of stay at the refugee camp; however, data concerning the individual duration of stay were only available for the Celle cohort.

Our study revealed that acute respiratory infections are the main cause for seeking medical care in the adult refugee population. Additionally, refugee patients with ARD have considerably more consultations than the nonrefugee German population. Although the study is based on data from the pre-COVID-19 era, it can be concluded that refugees are particularly affected by infectious respiratory diseases. With regard to COVID-19, refugees and migrants should be prioritized in vaccination campaigns, and regular testing should be offered to this vulnerable population.

### Reporting summary

Further information on research design is available in the Nature Research Reporting Summary linked to this article.

## Supplementary information

Reporting Summary

Supplementary Information

## Data Availability

The datasets generated and analyzed during the current study are not publicly available in accordance with the decision of the involved Research Ethics Boards but are available from the corresponding author on reasonable request within a data-sharing agreement.
