# Supplementary material for: Acute respiratory infections in an adult refugee population: an observational study
Source: NPJ Prim Care Respir Med. 2021 Dec 21;31:50. doi: 10.1038/s41533-021-00261-9 (PMC8692464; doi:10.1038/s41533-021-00261-9)
Supplement: Supplementary file 2 — Supplementary Information [file 41533_2021_261_MOESM2_ESM.pdf]

Supplement Material

Supplementary Table 1: Used definitions of respiratory diseases according to their ICD-10 codes.

**ARD**<br>(Acute infectious respiratory disease or signs of it)	**ILI**<br>(Influenza-like illness)	**Pneumonia**	**CRD**<br>(Chronic respiratory diseases)
• Cold (J00)<br>• Cough (R05)<br>• Sore throat (R07)<br>• Acute laryngitis and tracheitis (J04)<br>• Influenza (J11)<br>• Acute bronchitis (J20)<br>• Acute bronchiolitis (J21)	ARD<br><br>+<br><br>Fever (R50)	• Bacterial pneumonia (J15)<br>• Pneumonia, organism unspecified (J18)	• Bronchial asthma (J45)<br>• Chronic bronchitis (J41, J42)<br>• Chronic obstructive pulmonary disease (J44)<br>• Vasomotor and allergic rhinopathy (J30)

Supplementary Table 2: Factors influencing the rate of consultations with acute respiratory diseases or signs thereof: Multivariable analyses with age and occupancy rate as continuous variable.

Characteristic		Consultations with patients with acute respiratory diseases or signs thereof (ARD) (n = 1209)	
		Multivariable OR‡	p
**Sex#**	female	ref	
	male	1.03 (0.88-1.21)	0.695
**Age (continuous)**		0.99 (0.98-0.99)	<0.001
**Region of origin***	Europe & Central Asia	ref	0.007
	Latin America & Caribbean	1.72 (0.45-6.58)	
	Middle East & North Africa	1.04 (0.78-1.40)	
	South & East Asia	1.10 (0.78-1.54)	
	Sub-Saharan Africa	0.56 (0.37-0.83)	
	unknown/ stateless	0.84 (0.45-1.58)	
**Reception facility**	Celle	ref	<0.001
	Friedland	0.42 (0.33-0.53)	
**Occupancy of facility**		1.00 (0.99-1.00)	0.878
**Month of treatment**	January	ref	<0.001
	February	1.46 (1.10-1.92)	
	March	1.02 (0.73-1.42)	
	April	0.51 (0.33-0.78)	
	May	0.47 (0.26-0.84)	
	June	0.64 (0.35-1.17)	
	July	0.87 (0.50-1.53)	
	August	0.77 (0.50-1.20)	
	September	1.17 (0.89-1.53)	
	October	1.26 (0.96-1.66)	
	November	0.79 (0.57-1.10)	
	December	1.48 (1.15-1.92)	
**Refugee category**	asylum seeker	ref	<0.001
	resettlement	3.03 (2.34-3.93)	
**Chronic condition present**	no	ref	<0.001
	yes	0.45 (0.37-0.55)	

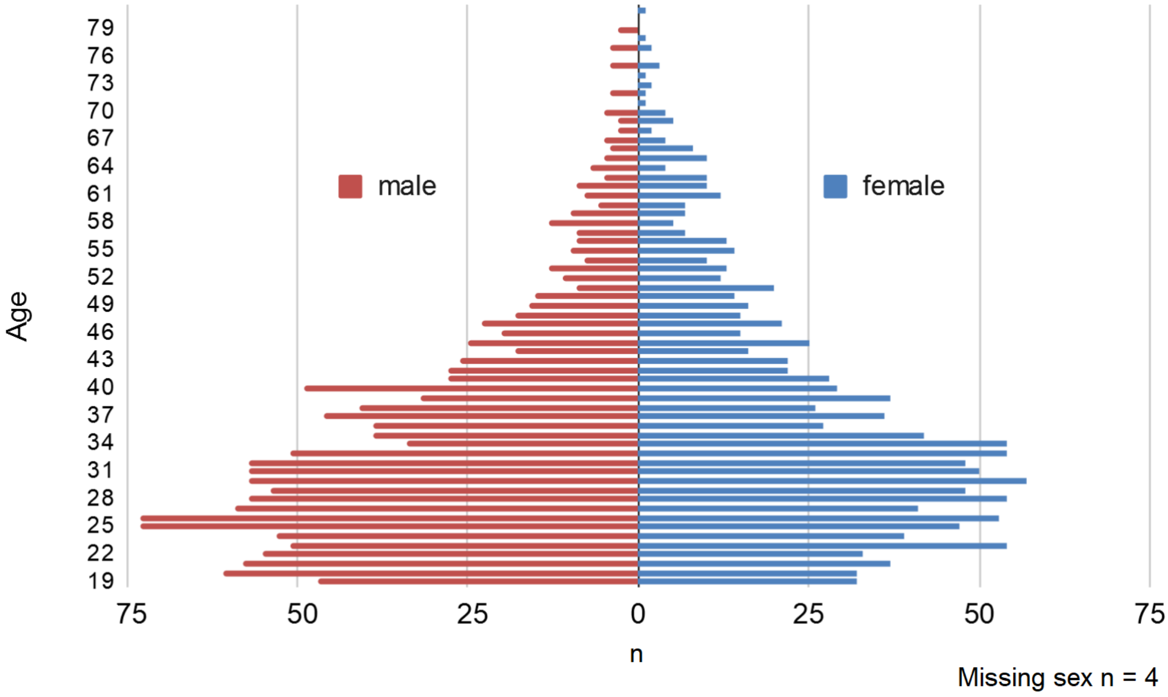

Supplementary Figure 1: Patients' age distribution (in years) by sex
